# Clinics and Churches: lifeworlds and health-seeking practices of older women with noncommunicable disease in rural South Africa

**DOI:** 10.1186/s12914-015-0051-1

**Published:** 2015-05-28

**Authors:** Daniel Lopes Ibanez-Gonzalez, Stephen M. Tollman

**Affiliations:** MRC/Wits Developmental Pathways for Health Research Unit, University of the Witwatersrand, Johannesburg, South Africa; MRC/Wits Rural Public Health and Health Transitions Research Unit, University of the Witwatersrand, Johannesburg, South Africa

**Keywords:** Lifeworld, Theory of communicative action, Healthcare, Access to, Healthcare, Remote/Rural, Healthcare, Users’ experiences, Health seeking, Illness and disease, Chronic

## Abstract

**Background:**

In this article we describe a phenomenological lifeworld study based on the theory of communicative action of 13 women with noncommunicable disease (NCDs) in a rural area in South Africa. The purpose of the study was to generate key concepts of health care access and the management of NCDs in a rural South African context.

**Methods:**

The study employed a qualitative methodology with serial semistructured interviews. We used a content analytical approach to analyse key themes and patterns in participants’ narratives of NCDs and health care access.

**Results:**

The findings are reported by theme and include analyses of narrative sequences related to 1) family environment, 2) experiences of NCDs, 3) understandings of the causes of NCDs, 4) accessibility of formal health care services, 5) experiences of formal health care services, 6) treating NCDs, and 7) experiences of informal health care services. The findings suggest that participation in the routines prescribed by formal health care services and reinforced by families and faith-based communities normalises the experience of NCDs to the extent that narratives of NCDs form the background, rather than the focus of broader illness narratives. Such narratives rather tend to focus on significant life events and relationships. The key features of the narratives include connections between social or autobiographical and biological understandings of NCDs, the appropriation of modern concepts of disease in illness narratives, and reflexive commentary on the modern features of NCDs. In the context of such narrative expertise formal health care services have a high level of acceptability in this rural area.

**Conclusion:**

Lifeworld analysis of health care access based on the theory of communicative action places consensual understandings of NCDs and their treatment as central to the health care experience. Our findings suggest that such analyses can facilitate potential feedback processes between health care users and professionals which generate consensus as well as institutional reform within formal health care services.

## Background

Although standard definitions of “rural” have been lacking in policy and research literature, one of the defining characteristcs of a rural area is its relative lack of amenities and infrastructure [1]. In rural South Africa, obstacles to accessing healthcare are often related to the long distances individuals have to travel, especially for specialist care [2]. The distance of healthcare services in remote rural areas has several implications for the quality of care, including long waiting times at the local community clinic during doctors’ visitation periods, shortage of medicines, long waiting time for emergency services, and long and arduous travelling conditions to far away secondary or tertiary medical facilities [2].

In these circumstances, it is often surmised that persons with noncommunicable disease (NCDs) in rural areas employ multiple health care paradigms to ensure affordable healthcare access for the treatment of their conditions [3]. The use of a variety of health care service providers, and the delayed use of modern allopathic health care services are prevalent themes in much of the health access literature in low- and middle-income and rural settings in Africa [4–8]. Such health-seeking practices are characterised by “healer shopping” (switching numerous times between healers), poor knowledge of allopathic or formal clinical diagnosis, and using formal health care facilities such as the clinic as a last resort after traditional or informal therapies are judged to have failed [9]. Elsewhere it has been argued that the strong reliance on traditional therapies and folk beliefs in rural areas has facilitated the transmission of disease, particularly HIV/AIDS [10].

This article explores how different health care resources might be utilised by women with NCDs in a rural setting. The approach is phenomenological, focusing on the experience of living with NCDs and accessing different types of care and support. We describe a qualitative study conducted with 13 women with NCDs in rural north eastern South Africa.

This paper forms part of a PhD by publication, and the findings are partially explained elsewhere with reference to the broader project. The overall aim was to describe the experiences of women with NCDs with regard to living with their illnesses and accessing health care in an urban and rural area. This would form the beginning of a description of the lifeworld in relation to illness and health care [11]. The current paper describes in detail the rural component of that study.

### Conceptual framework: Lifeworld-based health care research

Phenomenological studies of health care are characterised by an interest in the experience of health care, either from the perspective of health care providers [12], health care users [13, 14], or both, in the form of interactional studies [15, 16]. Phenomenological lifeworld research which has focused on the embodied experience of illness as expressed in illness narratives tends to focus on elements of narrative which shape meanings of illness [17, 18]. Such research is generally based on small sample sizes, as the focus is not on the representativeness of the views expressed, but rather on the processes by which meaning is constructed [12–18].

The lifeworld emerged as a conceptual unit of analysis in the phenomenological and hermeneutical traditions of the social sciences, and has often been posited in terms of the contrast between subjective experience and modern rational social systems [19]. More recently, the concept was systemised within the theory of communicative action as the cultural, social and personal interpretive framework through which an individual interprets and interacts with others in formalised social situations [19, 20]. Communicative action refers to the processes whereby individuals in social interactions arrive at consensual norms and reciprocal expectations regarding behaviour [20]. Such action has been posited as a moral imperative for social scientists researching social interactions [21], or as a goal worth attaining in its own right.

Lifeworld research focuses on such diverse aspects of the illness experience as physical and social space, physical impairment, pain and the role of narratives and strategy in shaping the relations between subjective experience and public accounts of illness [15–18]. Often the relationship between the subjective experience of illness and public accounts is expressed in terms of conflict and intrusion upon private space, as indicated in the standard term used in this connection: “colonisation of the lifeworld” [20]. Life world colonisation is particularly salient in health care settings which are characterised by regular encounters of individuals with deeply-held personal understandings of their bodies and their illnesses and health care personnel who are often trained to focus on disease in terms of technical biomedical discourses. Studies have shown, however, that lifeworld colonisation is not a uniform process and that the nature of the interactions between health care users and health care providers vary [15], indicating that the term may be tendentious. The current study focuses particularly on the experiences of health care users in living with NCDs and accessing formal and informal health care services.

### Methods

This study addressed the question: “How do women with NCDs experience their illness and access health care in a rural South African setting?” We answered this question with a qualitative methodology, using serial semistructured narrative interviews with women with NCDs.

### Study Setting

The study was conducted in the Agincourt sub-district in northeast South Africa, which at the time of fieldwork consisted of 90 000 people residing in 16 000 households in 27 villages [22]. The settlement patterns of the study setting were typical of rural communities across South Africa, particularly within areas (“homelands”) previously demarcated for black inhabitants of South Africa under the *apartheid* regime. Being itself located in a former homeland, the study setting consisted of a number of densely populated villages surrounded by fields used for grazing and harvesting of natural resources [23]. Infrastructure within the subdistrict was limited, although undergoing development. The area was marked by an absence of formal sanitation, erratic supply of piped water to communal standpipes, gravel roads and limited electricity supply. Local employment and farming was limited, with most adults seeking work elsewhere. There was one health centre and five satellite clinics within the site, and three public hospitals within 60 km of the site [24].

### Study Sample

Our sampling strategy was shaped by the study’s theoretical and institutional context. Theoretically, the study was undertaken as part of a larger comparative study [11]. Because we were interested in isolating cultural aspects of the rural lifeworld in relation to health care, we purposively selected villages which were comparable to urban areas in terms of the availability and affordability of formal health care services [25]. In case study research this is referred to as a “most similar” case study design, which entails the exploration of outcomes of theoretical interest (features of the lifeworld) where both cases share similar factors (availability and affordability of formal health care services) [26].

The selection of villages which would best suit the theoretical concerns of the study was facilitated by the institutional setting. In particular, the study was nested within the MRC/Wits Rural Health and Health Transition Unit in Agincourt (the Agincourt research unit), and it was in consultation with members of this unit that “most similar” villages (to urban settings) were selected and a sampling frame produced.

The villages selected for the rural case study were Agincourt, Cunningmoor A, and Kildare A. Each village had access to basic facilities such as schools, churches, businesses, communcal water taps and formal health care services in the form of a clinic within the village. There were no major cultural or infrastructural differences between the villages.

The sampling frame within the three villages consisted of 28 women who had participated in the 2006 INDEPTH-World Health Organisation Study on Global Ageing and Adult Health, and who had indicated the presence of one or more long term illnesses, including arthritis, stroke, angina, diabetes, chronic lung disease, asthma, depression, hypertension, cataracts, and loss of teeth [27].

The focus on women was guided by both pragmatic and theoretical considerations. In both urban and rural components of the broader study, the sampling frame overwhelmingly consisted of female heads of households. Theoretically, the study was definitional, and so we were interested in views which could be delimited to comparable groups of individuals in both study sites who could be considered expert-consultants in the illness and health-seeking aspects of the lifeworld. Such expertise was inferred by virtue both of their NCD status and their experience in accessing health care systems on their own behalf and on behalf of their family members [28]. It was in the interest of delimiting the parameters of our investigation and reducing potential complexities in data collection and analysis that we chose to focus on NCDs as the illness experience, rather than HIV/AIDS, or infectious disease. Although the health transition in South Africa has been characterised by the simultaneous occurrence of all these diseases, the rise in NCDs and associated risk factors is predicted to place increasing burdens on South Africa’s health care system [29].

We employed a purposive sampling technique to ensure sufficient variation in terms of village of residence and that we interviewed women with both single and multiple cases of NCDs. Within a lifeworld approach to the study of health care practices, study participants are consulted as experts by virtue of their experience, rather than subjects with representative views [14, 16–18]. Sample size is thus not determined by the extent to which the views of participants represent those of a broader population, but by the extent to which the data gathered reveals key features of the process of constructing meaning within a particular segment of the lifeworld.

In the current study data collection and analysis occurred concurrently, with each process informing the other, and data collection proceeding until a “saturation point” had been reached (referring to the exhaustion of conceptual categories for identifying key observations) [30]. These conceptual categories form the framework for reporting on key features of the lifeworld in relation to the experience of NCDs and accessing formal and informal health care services. We completed a total of 25 interviews with 13 respondents.

### Ethics

We discussed with each participant the purpose of the study: namely to investigate the experience of NCDs and accessing health care in a rural area. All personal information is kept confidential. Each participant gave her informed consent by signature to participate in the study and to have the interviews recorded and the recordings kept by the researcher for a period of 6 years. The research was approved by the Human Research Ethics Committee (Medical) of the University of the Witwatersrand (M090235).

### Data Collection

We collected the data between July and September of 2010, during which time 2 interviews were conducted with each respondent, with the exception of the last respondent who was unavailable for the second interview. The serial methodology allowed us to refine the initial coding categories during the second interview. We interviewed each respondent in her home, usually in a quiet spot in the garden and beneath a tree. The interviews were conducted in Shangaan by a local research assistant. The principal investigator (first author) drove the research assistant to her interview appointments and discussed with her the mood and content of the interviews after each interview. After the interviews for the day had been completed, both research assistant and principal investigator compiled detailed field notes on each interview, and identified key themes for further investigation. We recorded each interview, which was later transcribed and translated by the research assistant. An independent reseach assistant at the Agincourt research unit who was fluent in Shangaan checked a sample of the translated transcripts and found a high level of accuracy.

Our choice of narrative enquiry as the primary methodology required us to view the interviewee as a narrator with a unique life history [31]. As a result, we designed the interview guide to invite life histories around specific encounters, focusing on descriptions of NCDs, initial reactions to disease, experiences and observations of formal and informal health care services, and the impact of NCDs on daily life.

### Data Analysis

We employed a content analytical approach to data collection and analysis, and examined key themes and patterns in participants’ accounts of NCDs and health care. We derived the coding categories directly and inductively through the raw data [30]. We derived the initial coding categories during data collection via the analysis of detailed fieldnotes. After data collection, we hand-coded the transcripts using an open-coding technique to sort through the data and develop additional coding categories. An independent scientist not connected with the study coded a sample of the transcripts and we found a high level of agreement in our coding. The coding processes resulted in the following broad coding scheme:

1. Family environment;
2. Experiences of NCDs;
3. Understandings of the causes of NCDs;
4. Accessability of formal health care services;
5. Experiences of formal health care services;
6. Treating NCDs; and
7. Experiences of informal health care services.

We compiled and integrated all of the coded text from the 25 interviews into a single document including all coding categories. The document went through several iterations during which we refined the coding categories into a concise summary of the findings.

## Results

The findings begin with contextual findings related to family support, and continue to describe experiences and explanations of NCDs, access to and experiences of formal health care services, self-managed treatment of NCDs and the use of informal health care services. The 13 participants were between 55 and 90 years of age, and described a combination of NCDs, with hypertension being a common complaint. One participant reported not taking any form of formal or allopathic medicine, and eight participants reported using some form of informal or complementary medicine (including prayer) in conjunction with formal medicine. All but one participant lived with relatives or family members [Table 1].

**Table 1:** Summary description of study participants (N = 13)

Interview	Village Name	Age	NCDs	Treatment	Cohabiting primary support
A	Agincourt	79	Arthritis	Clinic medication	Daughter-in-law
B	Agincourt	63	Arthritis and ulcers	Clinic medication	Husband
C	Agincourt	90	Hypertension	Clinic medication and holy water	Daughter
D	Agincourt	55	Hypertension and ulcers	Clinic medication, diet and holy tea	Daughter
E	Cunningmore A	80	Hypertension and diabetes	Clinic medication, diet and holy tea	Co-wife
F	Cunningmore A	67	Hypertension, diabetes and arthritis	Clinic medication and diet	Living alone
G	Cunningmore A	89	Hypertension, diabetes and ulcers	Previously traditional medicine, now clinic medication, prayer and diet	Grandchildren
H	Cunningmore A	82	Hypertension and diabetes	Clinic medication, physical activity, prayer and holy tea	Daughter and grandchildren
I	Cunningmore A	60	Hypertension	Clinic medication and diet	Daughters
J	Cunningmore A	63	Hypertension and arthritis	Clinic medication and diet	Daughters
K	Kildare A	63	Hypertension and arthritis	Clinic medication and home remedy	Husband
L	Kildare A	61	Hypertension and arthritis	Previously clinic medication, now physical activity, diet and home remedy	Husband
M	Kildare A	70	Hypertension and poor vision	Previously traditional medicine, now clinic medication and home remedy	Daughters

### Family environment

Generally, the family was the first to be informed of the first signs of NCDs. In all cases, the family recommended either the clinic or the doctor. With one exception, all respondents stayed either with their children, their grandchildren, their husbands, or their relatives. Living with the family ensured that they had someone to talk to about their illness, to transport them to the clinic or hospital, to promote their interests in interacting with the clinic, and to help them with household duties. Only one respondent, whose husband had recently passed away, lived alone. The implications of living alone, as she understood them, shed light on the important role played by immediate, cohabiting family members in alleviating the burden of NCDs:

The sugar [diabetes] started together with high blood. So the stress, I think it started the time I lost my husband. I think it’s because I’m always thinking. As you can see I’m staying alone, no one to talk to and no one to share anything, so I’m thinking that maybe they will attack me and kill me. Besides, as I’m having sugar diabetes what if it drops? Who will assist me? I’m always alone, and I have nowhere to go to, except on Sunday when I go to church. My child stays far away from me, so I think a lot (Interview F, age 67, Cunningmoor A, July 29 2010).

This study participant continued to receive financial support and regular visits from her son. She also regularly visited with one of her sisters, who advised her about which foods to eat and which to avoid. But her anxiety and fearfulness about living alone was prominent in the interview. She was advised by the nurses at the clinic to participate in the counselling services offered at the local clinic, but she preferred the counselling services of a clinic further away, and so she was saving up for the longer journey. The anxiety she felt about living alone was related to the development of her illness, a theme which we shall return to when we discuss the participants’ understandings of the causes of NCDs.

Whereas all the participants could refer to some form of care they were receiving from family members, a few spoke at greater length about their own caregiving roles within their families. One respondent spoke about her responsibility for monitoring her grandchilren, whom she had cared for since her children passed away:

When they are ill I always take them to the clinic and make sure that they take the pills accordingly. And if I see that my grandchild is taking the pill and alcohol at the same time I will shout at him because it is not good (Interview H, age 82, Cunningmoor A, August 02 2010).

### Experiences of NCDs

Respondents described their condition as “high blood”, “dizziness”, “headaches”, “not having power”. “sugar diabetes”, and “stress”. The impression we gained from the interviews was that most respondents were not greatly inconvienced by their illness, although most of them were using part of their pension money to employ people to help them farm and perform domestic duties, particularly washing. None of the participants appeared uncomfortable during the interviews. The chronic conditions imposed some inconvenience, but were not in themselves major causes of distress. Consequently, their illness narratives were devoid of emotive or dramatic terms. Rather, their experiences were expressed in prosaic language.

A respondent might answer the question “What is your condition?” with the words “I am fine, it’s just that I was not feeling well”, (Interview I, age 60, Cunningmoor A, August 02 2010), spoken by someone with high blood pressure. Another respondent, who had arthritis, ulcers and skin sores, said: “I can say that I am not that much ill exactly”. This particular respondent went on to describe her first reactions to her conditions by saying “I didn’t go anywhere and I didn’t do anything about it”, and when she consulted her husband, he told her that “it’s just the illness of nowadays”. She went on to say “I agree with him, but I ended up going to the doctor because I was feeling the pain” (Interview B, age 63, Agincourt, July 26 2010).

From this narrative, as well as others, it appeared that the experience of NCDs in and of itself was not characterised by great anxiety. Another respondent who had been diagnosed with high blood pressure, and who had been experiencing headaches and sore legs said “I’m fine. Even the high blood is not high nowadays”. She attributed her mild condition to the good advice she had received during a home visit by professional health care workers:

They advised me to avoid stress. They also told me to keep myself always busy for my health and to always drink one liter of water. So I’m doing as they told me and it has helped me (Interview L, age 61, Kildare A, August 6 2010).

In this case, the mild experience of NCDs was attributed to the success of a personal health regime initiated by a home visit, but sustained by a desire to avoid a lifelong dependence on formal medication, for she went on to say: “I ended up leaving the pills because I was training myself”. This portion of the narrative describes a redemptive moment initiated by the home visit because the health care workers at the clinic had previously told her that she would have to take formal medication for the rest of her life. She said that they told her: “as I was taking the pills then, so I would take them for the rest of my life. So it is not like that because I’m not taking them now” (Interview L, age 61, Kildare A, August 6 2010).

### Understandings of the causes of NCDs

Respondents attributed their NCDs to naturalistic causes, including occupational, dietary and social causes. Occupational causes include strenuous work done during one’s youth, as with one respondent, who said, “I was farming a big farm alone and baking bricks on my own, so that is why my back is painful” (Interview H, age 82 Cunningmoor A, August 02 2010). In speaking of dietary causes of NCDs respondents observed the changing nature of diets as a consequence of modernisation. The same respondent quoted above, who had been diagnosed with high blood pressure and diabetes, said during her second interview: “We don’t eat vegetables and other food that we were getting from the farm. Nowadays we are used to buying food” (Interview H, age 82 Cunningmoor A, August 23 2010). Another respondent expressed herself as follows:

I was always listening to the programme on television. Sometimes they interview the doctors about the illnesses of nowadays. You find that sometimes we create illnesses on our own, by eating Rama [margarine], or meat. . . . So you find that all these foods are not good for you. Sometimes they will say you must avoid cooldrinks, sweet things . . . and if you are following you will be better (Interview L, age 61, Kildare A, August 06 2010).

This respondent was diagnosed with high blood pressure, a condition which she associated with the “illnesses of nowadays”. This term was used in an earlier excerpt from interview B in Agincourt village, where the respondent’s husband reassured her that her symptoms were “just the illness of nowadays.” This phrase appeared on one other occasion during the course of our interviews, in interview H quoted above, when the respondent said of her own parents’ health: “I can say that they died a long time ago, but at that time there was no illness of high blood. I think that high blood is the illness of nowadays” (Interview H, age 82, Cunningmoor A, August 02 2010).

Narratives indicating social causes of NCDs have already been referred to in the previous discussion on family environment. Just as family life provided the greatest support for participants with NCDs, it was also a source of anxiety and stress, particularly when a relative or family member passed away. For example, one respondent attributed her symptoms of dizziness and fatigue to the stress of caregiving: “if you are a mother everything that is painful, it will pass by you”. But upon further probing, she explained: “when my relatives die it becomes painful to me, so I think that is the reason” (Intervew B, age 63, Agincourt, August 24 2010). The stress brought about by the death of children brought the added responsibility on elderly caregivers of caring for and regulating the behaviour of grandchildren. One respondent, for example, attributed her high blood pressure to “thinking too much” about her grandchildren:

I’m thinking too much because of my grandchildren. You find that I don’t have anything to help them. I do get the pension, but I can’t get by with that only, so I’m always thinking. That is why I know that the cause of the high blood is because I’m thinking too much (Interview H, age 82, Cunningmoor A, August 2 2010).

Four respondents had lived through the death of children, which brought added pain to the stresses of life. One respondent said: “if you have lost your children things are difficult. I have some [children left], but I am not satisfied” (Interview K, age 63, Kildare A, August 06 2010).

### Accessibility of formal health care services

Our study design was intended to focus on health care access in terms of acceptability, rather than affordability and availability. We selected villages with clinics. Clinical health care services were free of charge and within walking distance of the homes of the study participants, and therefore no concerns were raised during the interviews regarding the general accessibility of the clinic. In terms of the acceptability of formal health care services, the clinic or hospital was usually the first point of call. Diagnosis and treatment for either high blood pressure or “sugar diabetes” was described as a straight-forward affair, with little variety in experience. One respondent summarised the general experience: “I first consulted the clinic. I was given the pills and instructed to take them on time and the right dose. So I’m doing like that even now” (Interview I, age 60, Cunningmoor A, August 02 2010).

Neither in the course of the illness narratives, nor in response to direct probing were significant problems or negative experiences at the clinic raised. It did emerge however that although the clinic is often the preferred health care option, this is not always the local clinic. Several respondents described travelling great distances to clinics or doctors in other villages or the nearest town. This has already been alluded to in an earlier excerpt in the discussion related to the family environment. The respondents who described using health care services further away explained their decisions in terms of looking for better services with regard to staff attitude, availability and expertise.

Another common feature emerging from narrations of the first access of the clinic was the cross-referral. From the first presentation at the clinic with a physical ailment, respondents described being referred to the local hospital for diagnosis, a process which often required a stay of some days at the hospital. Following the diagnosis was the course of treatment, which was described as a standard process of collecting medication. Because this was a standard process, it could be carried out more easily for the patient at the local clinic. It was left to the patient to request a referral back to the local clinic.

### Experiences of formal health care services

We asked the respondents a series of questions designed to elicit narratives about their experiences at the clinics. The questions included: “what is a typical visit to the clinic like?” “What are the problems you experience?”, and “How are patients treated at the clinic?” Respondents described going to the clinic, collecting pills and going home, without much interaction with the clinic staff or other patients. When the narratives did turn towards interaction with clinic staff, they were generally characterized by expressions of sympathy, appreciation, and the tendency to excuse any shortcomings which might have been observed. One respondent said: “At the clinic they are taking good care of us. What I have seen is the shortage of nurses: one person must do a lot of work” (Interview F, age 67, Cunningmoor A, July 29 2010).

In other narratives, these expressions of sympathy were connected with a sense of etiquette which prohibited gossip at the clinic or about the clinic. One respondent said:

…if some one has a problem with a nurse or something at the clinic they just write their concerns and put it in the suggestion box…. We are not telling each other what happened. No. We just write if we want (Interview J, age 63, Cunningmoor A, August 02 2010).

Informal discussion regarding any perceived shortcomings was regarded as spiteful because, in the words of one respondent: “it will be like we want them to be fired” (Interview K, age 63, Kildare A, August 06 2010). This indicates a sense of kinship or community with the nurses at the clinic.

The reticence of interview participants regarding their experiences of formal health care services can also be explained by the uniformity of those experiences. One respondent described the process:

What they do at the clinic, they check the BP [blood pressure]. So they will tell you if it is high or low. If they find that it is high they will give you the small pill. After a few minutes they will recheck, and if they find that it is still high they will give you the same pill. Then you go home (Interview J, age 63, Cunningmoor A, August 02 2010).

In our small sample of interviews, we could detect no point of view which challenged the established diagnosis and treatment procedures offered at the clinic. Rather, the general acceptance of the clinic as part of the experience of NCDs meant that the the overall experience was marked by regular and formal interactions which seemed to provide a sense of stability for the participants. For example, the respondent quoted in interview J above regularly went to the clinic and took her medication. She also reduced her sugar intake, in accordance with advice received at the clinic. Neither she, aged 63 at the time of the interview, nor her mother, who had passed away three years earlier, used home or traditional remedies for the treatment of their high blood pressure.

### Treating NCDs

We have seen in the discussion of experiences of NCDs the description of one respondent who had stopped taking pills because she was training herself. A different part of the same narrative was excerpted in the discussion of understandings of the causes of NCDs, where the respondent attributed her high blood pressure to modern diets. She went on to describe a life change from relying on daily medication, to a proactive approach including physical activity, drinking one litre of water per day, abstaining from soft drinks, sugar and condensed milk, and regularly drinking aloe tea. This allowed her to stop taking pills to control high blood pressure, which she felt able to control by her new lifestyle (Interview L, age 61, Kildare A, August 06 2010).

For the most part, however, respondents described a treatment schedule which was compliant with the instructions they had received from the clinic. One respondent said:

I don’t use any home remedy. I don’t even know how to mix it. Even if you can explain to me how they mix I will forget, because I’m not used to it. What I know is the medicine from the clinic. . . . I’m not educated, but I know how to take the pills and how they are working (Interview H, age 82, Cunningmoor A, August 02 2010).

This respondent was very clear about relying only on formal medication, saying “if you concentrate on the pills you will live”. However, her faith in the efficacy of the medicine from the clinic was in itself cited as a resource for getting better. She went on to say: “Even if you just drink medicine, if you don’t believe you won’t get well. So the only thing is to believe in what you are doing”. Faith and a proactive view of living with her illness formed her core belief with regard to coping with NCDs, while medication from the clinic played a supportive role. She went on to say:

Even if I can show you my card you will see that my high blood is not too bad because I can control it every time. I laugh with people to avoid thinking, or take a hoe to clean the farm. I can’t sit still because I know that I will start thinking, so I always do some jobs to avoid stress (Interview H, age 82, Cunningmoor A, August 02 2010).

### Experiences of informal health care services

Home remedies refer to herbal supplements based on folk knowledge of local herbs and their properties, and which might or might not be prescribed by a traditional healer. Traditional medicine refers to medicine, including home remedies, which is specifically prescribed by traditional healers who base their therapies on powers of divination [32]. In their eagerness to dissociate themselves from traditional healers, some respondents would also deny the use of home remedies, although they would later mention taking some form of informal medicine based on their own knowledge of treating disease.

The strong disavowal or disapproval of traditional medicine seemed to stem from church membership, particularly of African Christian Churches, although the high cost of traditional healers was also mentioned as prohibitive, at least in terms of treating NCDs. One respondent, when asked whether she used any home remedies said: “I don’t want any [such] medicine because I’m a Zion”, (Interview C, age 90, Agincourt, July 27 2010) referring to her status as a Zionist Christian. At the same time it was clear that she took some kind of informal medicine in the form of holy water, which did not replace medicine from the clinic, but which was held to assist in the treatment of NCDs. Other respondents followed the same practice.

Another respondent explained in more detail how her church membership placed restrictions upon the use of traditional medicine:

I was consulting the traditional healer because of the illness, but since I’m now going to [the Pentecostal] Church I no longer consult the traditional healer (Interview G, age 89, Cunningmoor A, July 29 2010).

As a member of the congregation this respondent was expected to abstain from consulting traditional healers. Her membership of this particular church placed her at odds with members of her family, who still consulted traditional healers, but, she explained: “I have withdrawn from the traditional healer. I can’t go there” (Interview G, age 89, Cunningmoor A, July 29 2010). Her church “said that we must concentrate on the pills only”, although ultimately health came from spiritual practices. She said: “If I was not praying I don’t think that I would be here now, so I’m praying” (Interview G, age 89, Cunningmoor A, July 29 2010).

Some respondents admitted to consulting traditional healers, but, in these cases, the consultation was not in relation to their NCDs, but for other purposes. For example, one respondent, who was a member of the Zion Christian Church, said that in her youth she had consulted a traditional healer for the purposes of conceiving a child. The social expectation to have children proved stronger than either her or her husband’s commitment to their churches to avoid traditional healers. She said:

…if people say “go there” you will go to try. Even my husband, he was not consulting traditional healers because he was going to the Apostolic church, but he was the one who took me to the traditional healer for the child (Interview E, age 80, Cunningmoor A, July 29, 2010).

Another respondent was more reticent concerning her reasons for consulting a traditional healer. She said: “I’m not taking home remedy, but I do consult the traditional healer, but not because of high blood. It is because of something else” (Interview I, age 60, Cunningmoor A, August 02 2010). It is probable that the primary motivation for consulting traditional healers in this region is to address one’s social relations with family members or neighbours. For example, one respondent admitted to consulting a traditional healer due to her suspicion that she had been “poisoned” by an envious neighbour:

Respondent: I consulted the traditional healer because I ate something like poison.

Interviewer: What have you eaten?

Respondent: It is like when some one gives you food and you don’t know if she has given it to you with pleasure or with bad intentions.

Interviewer: What did the traditional healer do?

Respondent: She removed it.

(Interview A, age 79, Agincourt, July 23, 2010).

## Discussion

This study began as an exploration of rural experiences of living with NCDs and accessing formal and informal health care services using the lifeworld approach. From the beginning we focused our data collection on the experiences of women in a rural area where formal health care facilities were both available and affordable, so that the acceptability dimension of health care access would form the core of our investigation [25]. It is evident from our findings that such a strategy proved successful: all our respondents lived in villages with clinics. The character of the narratives we encountered may be described as rural South African reflections on living with NCDs and accessing formal and informal health care services.

The question may be raised regarding the extent to which the villages selected were representative of rural areas in South Africa. Literature indicates that rural areas in South Africa vary substantially in terms of climate, environment, demography and infrastructure [1, 2]. The Agincourt sub-district, in which this study was conducted, has generally been accepted as a rural area, whose development, although perhaps more closely monitored than most areas in South Africa, has still been characterised as slow and uneven [22–24]. Besides these considerations, the use of a phenomenological lifeworld approach implies that the relevance of our findings to other contexts lies in the recogniseable and orderly features of narratives describing the experience of NCDs and health care access. The distinctly rural or South African features of these narratives emerge in relation to similar themes from similar contexts.

Within the lifeworld approach, we adopted the theory of communicative action which focused our attention on the concerns of individuals with NCDs in arriving at common understandings of their illnesses with family and community members, as well as with clinicians [19, 20]. This orientation may be contrasted with approaches to health care research which assume that health, rather than health care, is the primary concern of patients [33]. The theory of communicative action does not exclude the importance placed by individuals with NCDs on their health, but rather focuses on how such concerns are articulated in the context of regular social encounters.

All the research participants held broad understandings of NCDs which encompassed their social as well as their biological lives. We have seen, for example, how the family was acknowledged as an important resource in living with and accessing treatment for NCDs, but we have also seen how disturbances in family life, particularly the death of relatives or children, were spoken of as causes of illness. This raises the question of the extent to which traditional African concepts of health, which are relational in nature, as seen for example in the Basotho, persist in modernising contexts [34]. Such a relational concept of health, where the health status of the individual is inextricably linked with his or her family and community relationships, would be considered a feature of the lifeworld. It should be added, however, that these understandings of health and illness were not expressed in traditional terms, which in this region of rural South Africa historically consisted of an elaborate system of social taboos [35]. Rather, our narratives indicate that the relational concept of health and illness, particularly of disease causation, were pragmatic and consistent with modern discourses of disease which locate stress and anxiety as triggers of illness episodes.

The modern features of the narratives presented here relate to more than their content, such as the positing of naturalistic causes for NCDs; they also, in some instances, were evident as reflexive commentaries of the modern, or as perspectives in which the subject of experience was removed from her modernising context as a knowledgeable observer and commentator. This was most evident in the positioning of NCDs as “the illness of nowadays”.

It was clear from the narratives we examined that “the illness of nowadays” referred to NCDs, and was in one instance more specifically reflected upon as a disease of lifestyle, in this case, diet. The narratives suggest that this understanding of NCDs, by which they were related to the modernising environment of this rural area, formed part of a broader project which included adapting modern resources to rural lifestyles. For example, we have seen in our narratives that formal health care services had a high level of acceptance. Our respondents generally used either the clinic or the doctor as the first option in treating their NCDs. Switching of healers occurred largely within the paradigm of western health care, manifested as looking for better clinical services elsewhere.

The readiness with which participants in our study adopted modern naturalistic understandings of NCDs and corresponding formalised treatment regimens may be contrasted with other findings of qualitative studies of chronic disease. Elsewhere it has been argued that the bodily disruption brought about by NCDs brings the focus of individuals with NCDs on the minutia of their bodily functioning in the form of body narratives. In such a process, the use of informal medicine has been argued to enhance the process of forming coherent body narratives in which subjective control over the interpretation of bodily change is emphasised [36, 37]. Others have described the experience of NCDs in terms of existential chaos and confusion, characterised by a resistance to accepting the illness label and a sense of loss of one’s earlier life [38]. The narratives we have examined appear to tell a different story, in which straightforward or mild descriptions of NCDs are consistent with overarching narrative tones extending to descriptions of formal health care encounters as smooth social interactions characterised by camaraderie with the clinic staff and of medicine-taking as part of a familiar routine. The overall experience of NCDs was generally positioned as incidental to the real concerns of life, expressed as family concerns regarding the deaths of children or relatives, or the behaviour of grandchildren.

Such observations do not suggest that problems do not arise in accessing health care, or adhering to medical regimens. Rather, they emphasise a feature of social action which is often overlooked in public health discourse: namely it’s knowledgeable and order-sustaining character in relation to social institutions. This has been a subject of theoretical elaboration in modern sociological theory [39].

Finally, the narratives indicate that the accomplishment of situating NCDs within a modern and pragmatic context, both in terms of their causes and their treatment by formal regulated processes, is greatly facilitated by personal faith and membership of faith communities. We have seen how clinical treatment is reinforced by church-based healthcare practices, which are characterised by a disapproval of traditional medicine and worldviews as well as faith in the efficacy of formal clinical treatment. It appears as if churches and clinics share similar orienting functions within the larger project of the modernisation of rural areas, entailing the erosion of traditional worldviews. Whether this can be termed a type of colonisation of the lifeworld would require further investigation along these lines, but our own study suggests that a revision of terms commensurate with the practical agency of lifeworld subjects be explored.

## Conclusions

The limitations of our study have been alluded to in our paper. Primarily, these relate to the representativeness of our findings to rural contexts, and to the general experience of NCDs. It may be argued that our choice to focus on the generic experience of NCDs, as well as our focus on the social aspects of that experience have rendered our findings irrelevant in clinical settings, where it is assumed that persons with particular forms of NCDs are primarily interested in clinical endpoints [33, 40]. Our response to these potential objections has also been indicated in our discussion. Our study was not intended as a situational analysis of NCD prevalence or health care utilisation. Rather, the study was undertaken with the aim of defining health care access with reference to a lifeworld analysis of the most frequent users of health care services [11].

It may be argued that there is the possibility of social desireability biases in the findings, particularly in relation to the expressed approval of the clinic and disavowal of traditional medicine, especially since use of the latter is discouraged both by the church and the clinic. There is nothing within the narratives themselves, however, to suggest that this is the case. There is no reason not to accept the explanations for these preferences as evident in the narrations: the simplicity and regularity of clinical treatment, as well as its social acceptability, make it a natural preference in this context. It should also be noted that traditional healers were not altogether disavowed; rather, their relevance was confined to specific situations, whereas NCDs, which were naturalistically understood and accepted as a regular feature of life, required treatment approaches which were simple, inexpensive, regular, socially accepted and at least partially effective. Finally, it is clear that the clinic and clinical medication emerged in the narratives as suitable means of treatment within broader treatment strategies which included prayer, faith and a positive outlook. This deeper consideration of the findings suggest that they cannot be explained by recourse to social desireability biases, but rather indicate that they are best understood when accepted in their own terms.

Our findings indicate several broad themes related to the modernisation of spaces and lifestyles in South African rural settings, and point to potential structures of the lifeworld which accommodate these processes. With regard to clinical relevance, our assumption has been that the mutual recognition and articulation of meaning is just as valuable to individuals with NCDs as health outcomes. A consideration of our findings may contribute to public health discourse by stimulating a reflective process on the mutual expectations held by public health care officials and health care users of the role of formal health care services in managing NCDs. Lifeworld analysis based on the theory of communicative action is a means of ensuring that the experiences and goals of individuals with NCDs feedback into the review of such expectations [21].

Lifeworld rationalisation, or processes whereby institutional values are shaped by the lifeworlds of users and members of formal health care services, requires institutional space, time, personnel, and commitment. In consideration of our findings reported here and elsewhere [11] four factors emerge which can guide future processes of lifeworld rationalisation with regard to health care services and NCDs:

1. Individual processes of constructing health and illness narratives;
2. Interactions with formal health care systems;
3. Interactions with informal health care systems (including traditional and contemporary); and
4. The mediating role of social support systems (including family and faith communities).

The relationship between each component of this emerging model is characterised by the first component - the process of constructing health and illness narratives - as the central component which is shaped by various types of social interaction, as it in turn shapes individual responses to social obligations. Research which is directly connected with processes of lifeworld rationalisation can confirm the tenability of this model.
